# Cytochrome Expression in Breast Cancer Xenograft Mice after 12 Weeks of Treadmill Exercise

**Published:** 2018-05

**Authors:** Bang Sub LEE, Eun-Ju CHOI, Wi-Young SO

**Affiliations:** 1. Sports and Health Care Major, Korea National University of Transportation, Chungju-si, Korea; 2. Dept. of Physical Education, Daegu Catholic University, Gyeongsan, Korea

## Dear Editor-in-Chief

Breast cancer is the most common cancer and the second most frequent cause of cancer mortality in women in the United States (1, 2). Exercise reduces the risk of breast cancer. Therefore, moderate physical activity or exercise is important in breast cancer risk reduction (3).

Cytochrome P450 (CYP450) enzymes are heme-containing proteins that mediate drug biotrans-formation (4). CYP450 enzymes perform Phase I metabolism of xenobiotic drugs and toxins. There is a relationship between CYP450 and breast cancer (5), but there have been fewer studies of CYP450 and exercise in this disease.

Therefore, the purpose of this study was to determine whether breast cancer is differentially affected by CYP450 and exercise could play an important role in breast cancer reduced and CYP450 enzymes within this liver.

Fifteen female BALB/C nude mice (10 wk old) in Chungju-si in 2014 were randomly divided into control (n=5), exercise (n=5, 18 m/min treadmill for 30 min, 5 d/wk for 12 wk), and drug treatment (n=5, Taxol 16 mg/kg, 5 d/wk for 12 wk) groups.

This study was approved by the local university and all rules by Helsinki declaration were observed exactly.

The MCF-7 human breast adenocarcinoma cell line was used as a model of human breast cancer (ATCC; NIH: MCF-7, Catalog #HTB-22). Cells were maintained in RPMI-1640 supplemented with 10% FBS at 37 °C in 5% CO_2_. Cells were collected and suspended at 25 × 10^6^ cells/mL in a mixture of Matrigel (BD Biosciences; Chicago, IL: Lot #005002, 14.6 mg/mL). Mice were injected with 0.2 mL of the cell suspension for a final dose of 5 × 10^6^ cells. The cell suspensions were injected subcutaneously on the ventral side of the abdomen using a 23-gauge needle. Beginning approximately 14 d post-implantation, palpable tumors were measured with electronic calipers (Fowler Instruments; Newton, MA) across two perpendicular dimensions. Mice were randomly sorted into groups of three, based on tumor volume.

Livers were obtained, weighed, homogenized in 0.1 M potassium phosphate buffer, PH 7.4, containing 0.125 M potassium chloride, 1.0 mM EDTA, and a protease inhibitor mixture (Sigma), and centrifuged at 13000 gr for 25 min. The post-mitochondrial fraction was ultra-centrifuged for 45 min at 250000 gr to obtain microsomes. The pel-lets were suspended in 10 mM Tris-acetate buffer, pH 7.4, containing 0.1 mM EDTA and 23% glycerol and stored at −80 °C. Protein concentrations were measured using the bicinchoninic acid (BCA) protein assay kit (ThermoFisher Scientific, Rock-port, IL) with bovine serum albumin.

CYP450 protein levels in the mouse liver were measured by western blotting (6). Mouse livers were weighed and RNA was isolated by using RNA-Bee isolation reagent (Tel-Test, Friends-wood TX), according to manufacturer instructions. One microgram of total RNA was used for cDNA synthesis with the High Capacity cDNA Archive kit (Applied Biosystems, Foster City, CA), and real-time PCR was performed with SYBR^®^ Green PCR Master Mix (Applied Biosystems) and an Eppendorf Mastercycler Realplex PCR instrument. GAPDH mRNA was used as the normalization control. Primers for mouse P450s have been described by our laboratory (7). All primers were custom-synthesized on a 50-nmol scale, desalted and lyophilized by MWG Biotech (High Point, NC). Primers were diluted to 100 mM in deionized water and stored at −80 °C. All descriptive data were expressed in terms of mean±standard deviation. The Kruskal-Wallis test was used to examine differences between groups and post hoc test Tukey’s test using ranks was used to identify group differences. All analyses were performed in SPSS ver. 21.0 (SPSS, Chicago, IL, USA). Statistical significance was defined as *P*<0.05.

**Fig. 1:**
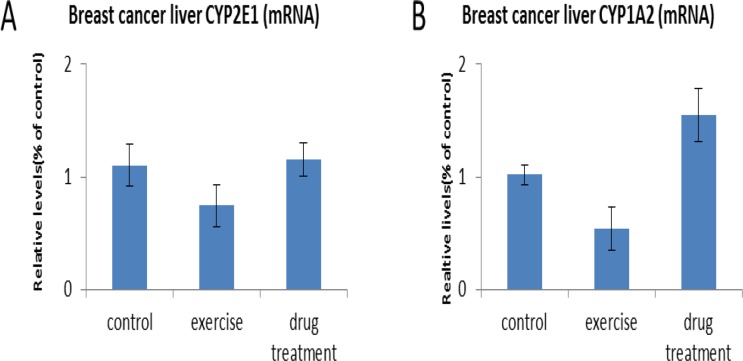
Transcript expression of (a) CYP1E1 and (b) CYP2A2 in breast cancer xenograft mice. There were no significant differences between groups (Kruskal-Wallis test)

**Fig. 2:**
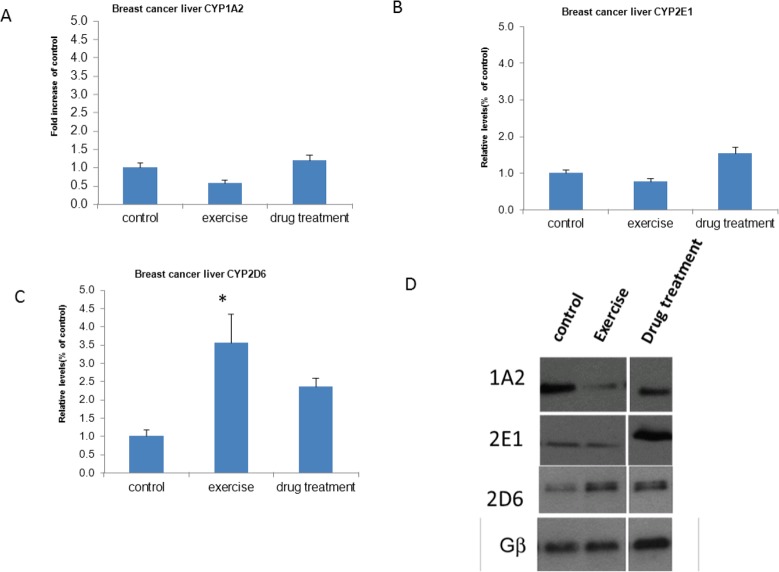
(a) CYP1A2, (b) CYP2E1, and (c) CYP2D6 expression in the hepatic microsomes of breast cancer xenograft mice; (d) representative western blots CYP2D6 activity was significantly greater in the exercise group than in the controls (*P*<0.05) using the Kruskal-Wallis test; *P<0.05 vs. the control group

Transcript expression of CYP2E1 and CYP1A2 did not differ between the exercise (0.75±0.49 and 0.54±0.50, respectively), drug treatment (1.15±0.38 and 1.55±0.63), and control groups (1.32±0. 32 and 1.02±0.23, *P*>0.05) (Fig. 1). Similarly, liver levels of CYP1A2 and CYP2E1 did not differ between groups (exercise: 55.84±13.74 and 75.69±16.62; drug treatment: 114.28±19.20 and 151.95±0.54; controls: 95.18±18.13 and 98.17±14.39, respectively, *P*>0.05). However, CYP2D6 expression was greater in the exercise group (79.06±28.03) than in the controls (28.71±6.63; *P*<0.05) (Fig. 2).

CYP2D6 expression increased in the livers of breast cancer xenograft mice subjected to exercise in comparison to untreated controls. Thus, exercise may help prevent breast cancer or improve therapeutic efficacy.
